# The Role of Biotechnology for Conservation and Biologically Active Substances Production of *Rhodiola rosea*: Endangered Medicinal Species

**DOI:** 10.1100/2012/274942

**Published:** 2012-04-30

**Authors:** Krasimira Tasheva, Georgina Kosturkova

**Affiliations:** Institute of Plant Physiology and Genetics, Bulgarian Academy of Sciences, 1113 Sofia, Bulgaria

## Abstract

At present, more than 50 000 plant species are used in phytotherapy and medicine. About 2/3 of them are harvested from nature leading to local extinction of many species or degradation of their habitats. Biotechnological methods offer possibilities not only for faster cloning and conservation of the genotype of the plants but for modification of their gene information, regulation, and expression for production of valuable substances in higher amounts or with better properties. *Rhodiola rosea* is an endangered medicinal species with limited distribution. It has outstanding importance for pharmaceutical industry for prevention and cure of cancer, heart and nervous system diseases, and so forth. Despite the great interest in golden root and the wide investigations in the area of phytochemistry, plant biotechnology remained less endeavoured and exploited. The paper presents research on initiation of *in vitro* cultures in *Rhodiola rosea* and some other *Rhodiola species*. Achievements in induction of organogenic and callus cultures, regeneration, and micropropagation varied but were a good basis for alternative *in vitro* synthesis of the desired metabolites and for the development of efficient systems for micropropagation for conservation of the species.

## 1. Introduction

Resent investigation of the World Health Organization (WHO) recorded increasing utilization of herbs in the medical practice [1]. The European market of medicinal plant preparations and remedies has been stimulated by the national policies for better exploitation of the advantages of the traditional and the alternative medicine [2, 3]. The short period of time from 1999 to 2001 marked a considerable increase in the medicinal plants trade in many countries: in the Czech Republic by 22%, in Turkmenistan by 100%, and in Bulgaria by 170%. Similarly increasing herbs use in the developed countries was recorded [4]. More than 25% of the British population is taking them regularly [5]. The business becomes more stable with sales income reaching 5 billion USD for the period of 2003-2004 in Western Europe; 14 billion USD in China for the year of 2005, and 160 million USD in Brazil for the year of 2007.

At present more than 50 000 plant species are used in the two major fields: the contemporary phytotherapy and the modern allopathic medicine [4]. World-wide, about 2/3 of those 50 000 medicinal plants are harvested from nature [6]. The share of the cultivated plants used in the pharmaceutical industry is quite small, yet, being only 10% in Europe [5]. The number of natural populations is decreasing progressively leading to local extinction of many species or degradation of their habitats [7]. According to the data of the Food and Agricultural Organization (FAO) at the United Nations annually the flora bares irretrievable losses which destroy the natural resources and the ecological equilibrium [8]. Four thousand to 10 000 medicinal species were endangered of disappearing during the last years [6].

Bulgaria is in the leading world positions in export of wild medicinal plants. Annual harvest varies between 15 000 t and 17 000 t. Half of them are collected in the mountains while 80% of them are exported [9–12]. Bulgarian medicinal plants are famous for their high content of biologically active substances. They are ranked in the first world positions considering quality which is due to the specific climatic and soil conditions of the country [13].

However, the constant expansion of herbs trade, the insufficient cultivation fields, and the bad management of harvesting and overharvesting have led to exhaustion of the natural resources and reduction of the biodiversity. To stop the violence against nature, efforts should be directed to both preservation of the plant populations and elevating the level of knowledge for sustainable utilization of these plants in medicine—traditional, alternative, and allopathic [4].

This great issue is in the focus of science which offers different decisions to solve the global problem. Cultivation of the valuable species in experimental conditions is one of the approaches. The latter refers to application of classical methods for multiplication by cuttings, bulbs, and so forth, as well as by biotechnological methods of *in vitro* cultures and clonal propagation for production of enormous number of identical plants. The micropropagation is considered to have the greatest commercial and iconomical importance [14]. The modern techniques are especially appropriate for species which are difficult to propagate *in vivo *[15].

Biotechnological methods offer possibilities not only for faster cloning and conservation of the genotype of the plants [14, 16] but for modification of their gene information, regulation and expression for production of valuable substances in higher amounts or with better properties [15, 17]. Application of integrative approaches to cultivation of plant systems could be a basis for future development of new, more effective, safe, and higher-quality products. Biotechnological methods are reliable and provide continuous sources of raw material and natural products for food, pharmaceutical, and cosmetic industries [17–19].

## 2. *Rhodiola rosea* L.: Importance and Characterization

*Rhodiola rosea* is one of the endangered medicinal species with outstanding economical importance determined by its roots and rhizomes (*Rhadix et Rhizoma Rhodiolae*—rose root) utilization in medicine and pharmacy for more than thousands of years.

Golden root extracts have various properties—adaptogenic, antitoxic, antihypoxic. They increase the body's nonspecific resistance and normalize body functions by stimulation of the own-body biochemical and functional reserves. Rose root preparations regulate metabolism, the functions of the central nervous system, the heart, and the hormonal glands [20–23].

The chemical composition of the extracts from the *Rhodiola* root and rhizomes was studied by East-European research groups mainly [24–30]. A decade of investigation [31, 32] revealed evidences about the presence of different biological active substances in the rhizome of golden root unlike some other *Rhodiola *species.

Six different groups of chemical components with pharmaceutical interest could be found in the roots and rhizomes: (1) phenylpropanoids—alcohol derivatives of the cinnamon acid and glycosides like rosavin (2.1%) [33], rosin and rosarin (which are classified under the general name of “rosavins”) [30, 32, 34–36]; (2) phenylethanoids—salidroside (rhodioloside) (0.8%), *p*-tyrosol [37–42]; (3) flavonoids—including rodiolin, rodionin, rodiosin, acetylrodalgin, tricin [32, 34, 43], and tannins 16–18% [44]; (4) monoterpenes, including rosiridol and rosidarin; (5) triterpenes, such as daucosterol and betasitosterol; (6) phenolic acids such as chlorogenic, hydroxycinnamic, gallic acids [29] and essential oils [35, 45, 46]. All these substances determine the specificity of the *Rhodiola* extracts. However, the roots have many other substances like phenolic antioxidant, including proanthocyanidins, quercetin, gallic acid, and chlorogenic acid [23, 47].

Rosavins (rosavin, rosarin, and rosin) and the salidroside or rhodioloside, as well as rodiolin, rodonizid, and roziridine are the most important, and are mainly used as active substances for production of medical preparations. Tyrosol is also a crucial active ingredient; though to a less extent than the other two standards. The rosavins complex is specific for *Rhodiola rosea* unlike salidroside which presents in other *Rhodiola *species and in some plants from other genera [29, 48].

One of the major results of the research is the detection of differences in the content of biologically active substances in the roots of *Rh. rosea* depending on their habitats [40, 42, 48, 49]. Investigation of rose root from different Bulgarian mountains areas indicated the highest amount of salidroside of 1.55% in Rila mountain sites, and, respectively, the lowest of 0.72% in Pirin Mountain sites [50]. The stem and the leaves contain less salidroside, while there is no substantial difference in the levels of polyphenols accumulation in the epigeous parts of the plant.

### 2.1. Taxonomy and Morphology

*Rhodiola rosea* L. (*Sedum roseum* (L.) Scop., *S. rhodiola* DC.), rose root, golden root (*Crassulaceae *family) is a species, valuable for the world gene fund of herbs (medicinal plants) distinguished among the other 200 species of *Rhodiola* genus and esteemed for its outstanding pharmacological importance and use.

*Rhodiola rosea* L. is a succulent herbaceous, perennial, and dioecious plant (having separate male or female plants) with a thick quite branched scaly rhizome (rootstock) with average weight of 70–400 g, but reaching 3.5 kg, too. Root and rhizomes have rose scent. Several shoots grow from the same thick root. The stem is straight, 10–30 cm in height [51]. The leaves are oblong, elliptic-oblanceolate, or obovate, entire. Inflorescences are corymbiform or capitates, the flowers unisexual, flowers are set from April to August. Propagation is vegetative or by seeds [52].

Golden root grows mainly in the cooler regions of the world—the Arctic, Scandinavia, North Russia, in the mountains of Asia (the Himalayas, the Altai, and the Ural mountains) and Europe (the Alps, the Pyrenees, the Carpathian Mountains), and in other higher mountainous regions (980 to 2000 m altitude) like those in Bulgaria (the mountains of Sredna Gora, Rila, Pirin, and the Balkan) [53]. This plant prefers the scree, grassy, or rocky slopes, from mountain to subalpine zone of heights up to 2280 meters altitude [54].

Intensive and unscrupulous exploitation of the natural habitats in many countries has resulted in extinction of the species in these regions [55]. This provoked undertaking of a series of nature-protecting measures like (1) cultivation under appropriate conditions, (2) protection of the populations in the protected territories, (3) including the species in the Red Books of rare and endangered plant species.

*Rhodiola rosea* is included in the Red Books of Republic of Buryatia AR, of Yakut ASSR, of Mongolia; “Rare and Extinct Plant Species in Tuva A Republic,” “Rare and Extinct Plant Species in Siberia,” in Great Britain—Cheffings & Farrell, in Finland—category “last concerned.” *Rhodiola rosea* in Bulgaria is under protection of the law for biological diversity [56]. Activities concerning the protection and the sustainable use of the plant are described by the Law for medicinal plants [57]. Some *Rhodiola rosea* populations are included in reservation areas in the states of the Commonwealth of Independent States (CIS) mainly. *Rhodiola rosea* is successfully cultivated in botanical gardens and centers for introduction in Russia (Saint Petersburg, Gorno-Altaysk, Novosibirsk, Irkutsk, etc.) [58], Finland, and Norway mainly [59].

In Bulgaria, like in the other countries, the number of natural resources of *Rhodiola rosea* is decreasing progressively. The restoration capacity of the wild-growing plants are quite limited due to the very low, both, seed germination (5–35%) and coefficient of vegetative reproduction [26, 60]. Plants need from 4 to 6 years for optimal development and maximum accumulation of biologically active substances in the rhizomes [21, 60].

These data determine the interest towards *Rhodiola rosea* for establishment of *in vitro* cultures as initial raw material for the pharmaceutical industry and for the plant conservation.

## 3. *In Vitro* Cultures of *Rhodiola rosea* L.

Despite the great interest to *Rh. rosea* and the wide investigations in the area of phytochemistry, one potential area—that of the plant biotechnologies, remains less endeavored and exploited in comparison to other medicinal species. At present there are many scientific groups in the world working on various medicinal plants like *Panax ginseng*, *Arnica montana*, *Rauwolfia*, and *Galanthus* [15–17]. *In vitro* propagation has gained distinguished success, and during the last years it reached large commercial scale, corresponding to the demands of the pharmacological industry [15]. In spite of that, there is not an universal protocol applicable to all species of interest. Usually schemes are established for a particular plant for specific purposes. However, accumulation of experience and knowledge in addition to the empirical approach is a good basis for elaborating desired schemes for the target plants.

Pioneer experiments on golden root *in vitro* cultures were initiated in the nineties of the last century [61–63]. Next further research was directed to examine the factors important for a successful establishment of callus and organogenic cultures which depends mostly on the interactions of the explant, the nutritious media, the phytoregulators, and the culture conditions on the plant genetic background. However, the optimal combinations of these factors could be determined experimentally for each specific case.

The choice of the explant type is a crucial factor for successful realization of the morphogenic potential of the isolated cells. The explant could determine the organogenic and the genetic stability of the progeny after cloning. This is the reason for the great number of explants used for initiation of *in vitro* cultures in *Rh. rosea*.

**Figure 1:**
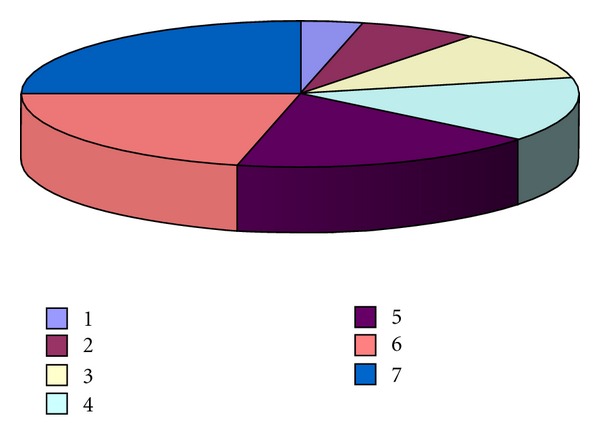
The relative shares of the most commonly used explants for initiation of *Rhodiola in vitro* cultures. Explant were excised from plants growing in their natural environment: (1) leaves, (2) stem, (3) shoot tips and buds, (4) nodes and rhizome buds, (5) axillary buds, (6) hypocotyl, and (7) apical buds and stem nodes from *in vitro* seedlings.

**Figure 2:**
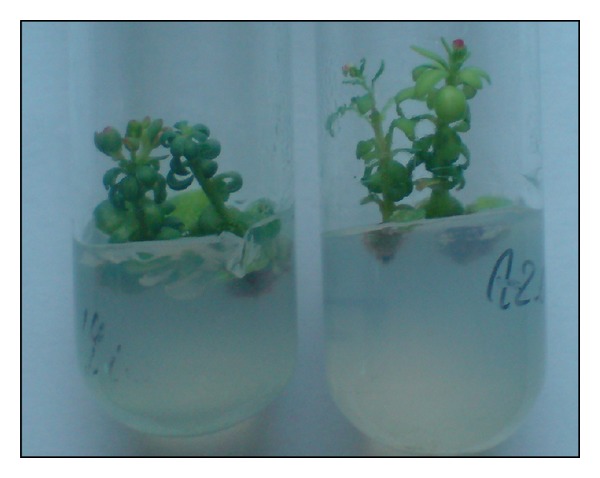
*Rh. rosea* plant regeneration from *in vitro* seedling apical buds on Murashige and Skoog (MS) [64] basal medium enriched with 2 mg/L zeatin, 0.2 mg/L IAA, and 1000 mg/L casein hydrolysate.

**Figure 3:**
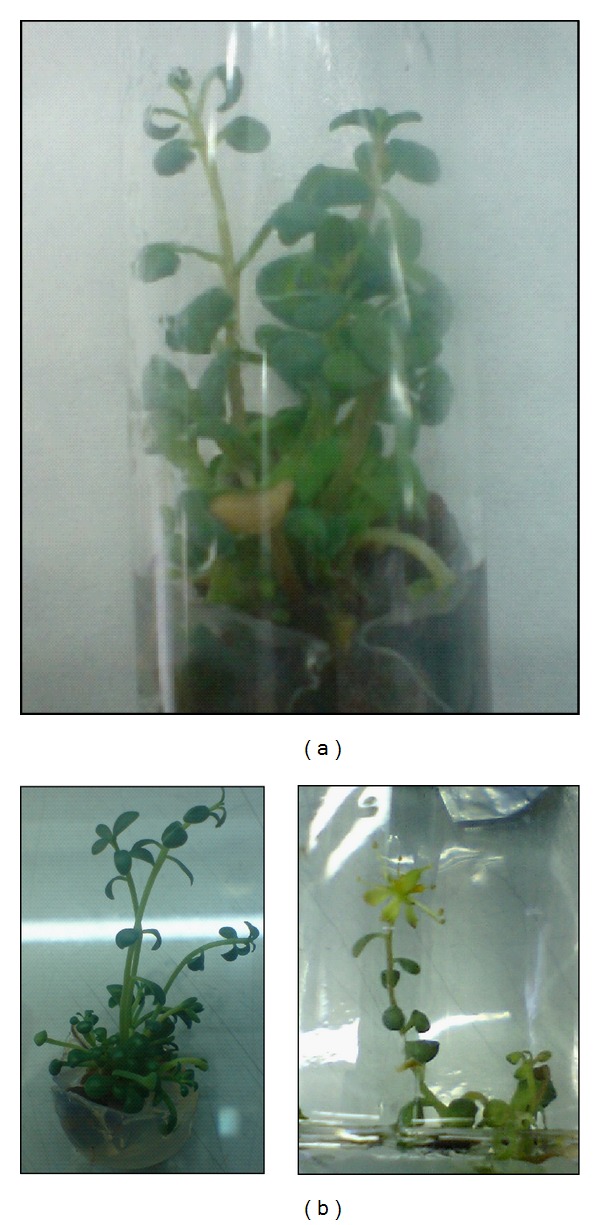
*Rh. rosea* plant regeneration from *in vitro* seedling leaf nodes (a); shoot multiplication (b) and flowering regenerant on MS basal medium enriched with 2 mg/L zeatin, 0.2 mg/L IAA, and 1000 mg/L casein hydrolysate.

Leaves or leaf disks were preferable explants for callus, bud, and shoot formation [62, 65–68]. Explants of less use were axillary buds [63], stem segments [67, 69], shoot tips and buds [69–71], and node and rhizome buds [69, 72] excised from plants growing in their natural environment and *in vitro* (Figure 1). Apical buds and stem nodes from *in vitro* seedlings were the object of others' investigations [69, 73, 74] (Figures 2 and 3). Obtained data reveal that experiments in the different laboratories have contradictory success.

**Figure 4:**
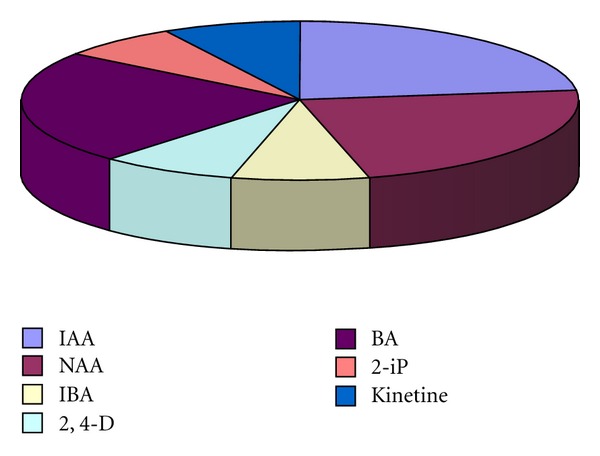
The relative shares of the most commonly used plant phytoregulators for *Rhodiola* callus induction *in vitro*.

**Figure 5:**
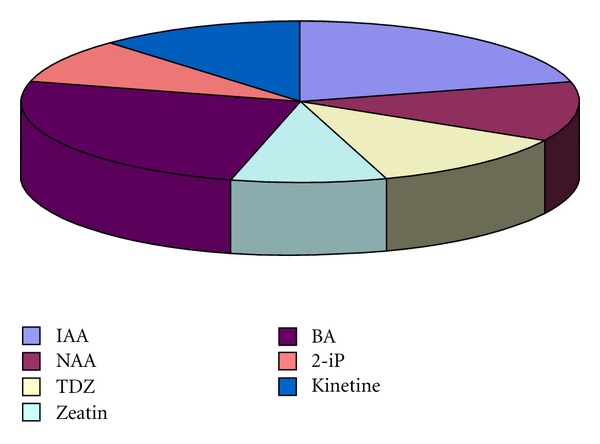
The relative shares of the most commonly used plant phytoregulators for *Rhodiola *bud and shoot induction *in vitro*.

**Figure 6:**
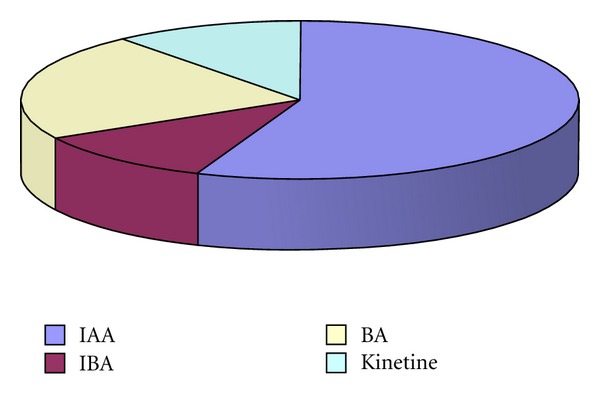
The relative shares of the most commonly used plant phytoregulators for *Rhodiola* root induction *in vitro*.

The nutritious media and the phytoregulators are the other very important factors influencing the processes of callogenesis, organogenesis, regeneration, and multiplication *in vitro*. The chemical composition of the media and its physical properties should correspond to the requirements of each stage of culture development [17, 75]. The test of different recipes of Murashige & Skoog (MS), Linsmaer & Skoog, Gamborg, White, and Nitsch & Nitsch showed that the last one was the best for plant development from shoot tips, while MS medium allowed various responses from different explants [65]. MS medium, compared to these of Litvay and of White, proved to be better for evaluation of morphogenic potential of *Rh. rosea* and *Rh. iremelica* [70] as well as for callus cultures and regeneration in Tibetan *Rh. rosea* [76]. The Murashige and Skoog medium became the most popular one for golden root *in vitro* cultures. Usually it is supplemented with various growth regulators in different combinations and concentrations. The most commonly used are 6-benzylaminopurine (BAP), indole-3-acetic acid (IAA), l-naphthyl acetic acid (NAA), indole-3-butyric acid (IBA), and 2,4-dichlorophenoxy acetic acid (2,4-D) [62, 65–67, 69, 70] but the effect of zeatin, dimethylaminopurine (2-iP), kinetine, and thidiazuron (TDZ) was examined, too [69, 71–73] (Figures 4, 5, and 6).

However, the efficiency of the plant growth regulators is in relation to the genetic background which was proven in the experiments with various *Rhodiola rosea* ecotypes [70]. The best results for revealing the morphogenetic potential could be achieved by the appropriate balance between the explant and the media enriched with phytoregulators and other nutritious supplements and the genotype. These are the three milestones in the experiments of each group aiming at establishment of *Rhodiola in vitro* cultures. Achievements in induction of organogenic and callus cultures, regeneration, and micropropagation varied, and some examples are presented in this review.

Leaf segments were used in one of the first investigations for induction of shoots and callus formation [62]. The response of this explant type was evaluated on quite a great number (15 variants) of Murashige and Skoog medium [64]. The importance of BA, both for dedifferentiation and redifferentiation processes was pointed out. This cytokinin in the absence of auxins caused adverse effect on leaf viability and callus formation. However, the ratio between its concentrations and that of IAA and NAA, determined induction of callogenesis and organogenesis. Callus and root formations was stimulated by isomolar levels of 5 *μ*M/L each BA and IAA while shoot formation was more efficient when the BA concentration was higher than the concentration of the auxin NAA 0.5 *μ*M/L.

Nearly at the same time quite detailed investigations were performed to study the interaction between two explant types and different nutritious media and growth regulators on the regeneration and callogenesis potential of *Rh. rosea* [65]. Using leaf segments and shoot tips different combinations of BA, Kin, IAA, 2,4-D, NAA and 2-iP were tested. The most favorable conditions for shoot formation were shoot tips cultivation for 8 weeks on Nitsch & Nitsch (NN) medium supplemented with 0.1 mg/L IAA and 0.1 mg/L or 1 mg/L kinetin. The same media favored root formation, too. However, addition of BA and IBA to NN medium led to lower levels of regeneration and to inhibition of the growth when NAA was included. Complete suppression of development of explants from leaf, hypocotyls, and shoot tips was observed on Linsmaer & Skoog medium containing 2-iP and IAA. Callogenesis was induced only by leaf segments plated on Gamborg-B5, Gibco, and MS media. However, adequate growth was observed on the last medium containing BA and NAA, or BA and IBA.

A different approach was used in other experiments [66]. Suitable plant material for initiation of shoot cultures was seeds which before *in vitro* germination were treated with low temperature of 2–4°C for a period of 4 and 6 months or with 0.02% GA_3_ for 24 hours. These procedures replaced successfully the seed stratification. Stem segments, stem apex, and leaf explants excised from the seedlings were cultured on MS medium enriched with BA and IAA in different ratio of concentrations from 1 mg/L to 3 mg/L of the cytokinin and from 0.8 mg/L to 2.6 mg/L of the auxin. The most efficient direct organogenesis was from leaf explants on MS medium supplemented with 2 mg/L BAP and 1.7 mg/L IAA.

Seeds and rhizomes from three ecotypes from the High Altai and South Ural region served as the explant source to study induction of callogenesis and organogenesis [70]. Explant development was observed on MS media containing various phytoregulators (BAP, IAA, NAA, IBA, 2,4-D). Vigorous callus formation of 86% of the explants was observed on MS medium enriched with 0.1-0.2 mg/L IAA. The optimal combination for multiple bud formation in *Rhodiola rosea *from stem segments was BAP and IAA in concentrations of 0.2 mg/L and 0.1 mg/L, respectively, while for* Rhodiola iremelica* the efficient concentrations were lower—0.1 mg/L BAP and 0.05 mg/L IAA. Differences in ecotype appeared to be also an important factor influencing the processes of efficient callogenesis and organogenesis. Regenerants were initially adapted in vermiculite for two weeks in conditions of high humidity (85–90%) and later plants were transferred to mixture of soil, peat, and vermiculite in proportion of 1 : 1 : 1. Plantlets revealed considerable differences (from 10% to 95%) in the survival rate during the process of adaptation. In the later experiments [76] the effect of *Rh. rosea* extracts on the morphogenic abilities of *Rh. rosea* and *Rh. iremelica* were studied. Addition of 5% or 10% v/v liquid extracts in the MS medium provoked different *in vitro* responses. Bud induction was stimulated by the lower concentration and inhibited by the higher ones leading to formation of 8.5 shoots from the explants in the first case and 1.1 in the second case.

In comparison to the previously described investigations with the Altai ecotype of *Rhodiola rosea* the optimal concentrations of the cytokinin BAP were 10–15-fold higher for induction of *in vitro* cultures from immature leaves explants from a Tibetan ecotype of golden root [67]. The authors note interaction between the growth regulators and the illumination of the cultures. Two mg/L BA and 0.2 mg/L NAA added to the MS medium stimulated formation of incompact callus tissue. However, when explants were cultivated under dark conditions, higher concentrations of the same phytoregulators 3 mg/L BA and 0.25 mg/L NAA were more efficient. MS medium containing 2 mg/L BA and 0.25 mg/L NAA induced shoot multiplication while rooting was induced on MS medium containing 0.5 mg/L or 1 mg/L IAA.

Recently [71] *in vitro* micropropagation of rose roots in a RITA bioreactor system was reported for the first time. Three clones were obtained from *in vitro* germinated seedlings of wild Finland golden root. The effect of two stimulators of organogenesis was studied. Two to four *μ*M thidiazuron stimulated shoot induction but inhibited shoot growth while 1-2 *μ*M zeatin favored increasing shoot size and leaf number per shoot. The clones differed significantly in respect to multiplication rate. One of them was distinguished for its high coefficient of shoot formation per explant on solidified medium enriched with 2 *μ*M zeatin. In the bioreactor thidiazuron maintained rapid shoot proliferation at quite concentration (0.5 *μ*M) but induced hyperhydracity at more than 0.5 *μ*M. However, hyperhydracity was abolished when shoots were transferred for 4 weeks on gelled medium enriched with 1-2 *μ*M zeatin. Shoots formed roots for 5-6 weeks on BM medium without phytoregulators. Most of the regenerants (85–90%) survived when transferred to soil. After acclimatization, the plants growing in the green house had normal shoot and leaf morphology.

An extensive research with Bulgarian *Rh. rosea* ecotype brought the development of a complete system for obtaining callus cultures, shoot induction and multiplication, micropropagation, and reintroduction of regenerants to the natural environment of the rose root. Success was based on the investigations of the interaction between numerous explant types, media supplements, and culture conditions. In the initial experiments [73, 74] apical buds and stem nodes excised from *in vitro* germinated seedlings/plants were used. Another series of experiments [69] examined the morphogenic potential of five types of explants obtained from wild-growing plants (apical buds, adventitious shoots, stem explants, rhizome buds, and rhizome segments). Different *in vitro* responses were observed—formation of plantlets, leaf rosette, various types of callus (compact green, pale, soft liquidy) and callus degeneration without bud formation. In another series of experiments the authors cultivated explants excised from 15–25 mm high seedlings—apical bud, stem segment with leaf node, and explants excised from the seedling root basal area. The stem segment with leaf nodes and apical buds were the best explants for *in vitro* propagation. Morphogenic potential was studied on MS basal medium containing a range of phytoregulator combinations, including basically benzyl adenine, kinetin, zeatin, 2-iP, 2,4-D, NAA, and IAA. Excised leaf nodes and apical buds from seedlings cultured *in vitro* proved to be a good solution in the investigations for overcoming the problems when working with wild plants. The both explants regenerated shoots with highest efficiency of more than 80% on zeatin containing media. The zeatin and the 2-iP, compared to kinetin and BAP, stimulated growth and formation of shoots from seedling leaf nodes and apical buds. The significance of zeatin for numerous bud/shoot formations was pointed out. It was interesting to note that the coefficient of propagation varied during the different seasons. Highest level of proliferation was observed in May-June, when the shoots per explant were 6.78. During the cold seasons multiplication was relatively lower—2.11 shoots per explant [77].

**Figure 7:**
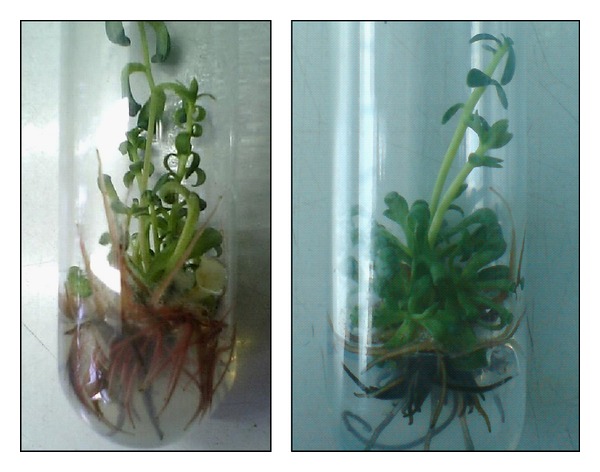
Induced rhizogenesis and propagation in* Rh. rosea *regenerated plants on MS medium enriched with 2.0 mg/L IBA, 0.2 mg/L IAA, and 0.4 mg/L GA_3_ [69].

**Figure 8:**
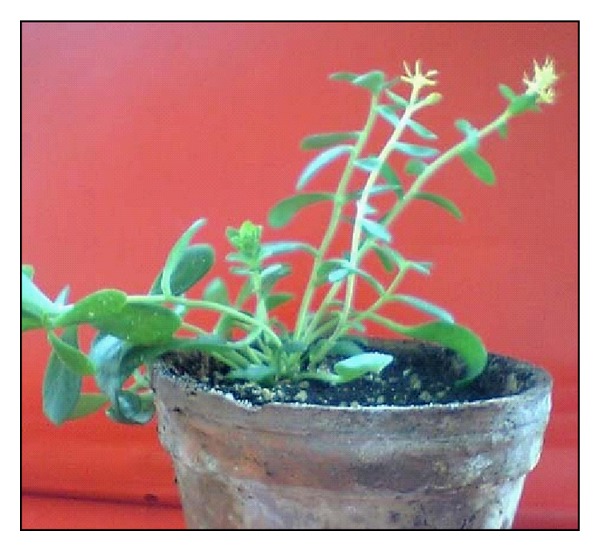
Adaptation of *Rh. rosea* regenerants in mixture of perlite, peat, and soil [69].

Rhizogenesis was stimulated by IBA in increasing concentrations up to 2 mg/L and was most efficient when IBA and IAA were applied in combination (Figure 7). The regenerants 3–5 cm in height which had 2-3 cm long roots were transferred to pots containing a mixture of soil : peat : perlite in a ratio 3 : 1 : 1 and were maintained for 20 days for adaptation in special chambers (Figure 8). Acclimatization of rooted golden root plantlets into the greenhouse was successful up to 70% [69, 77].

**Figure 9:**
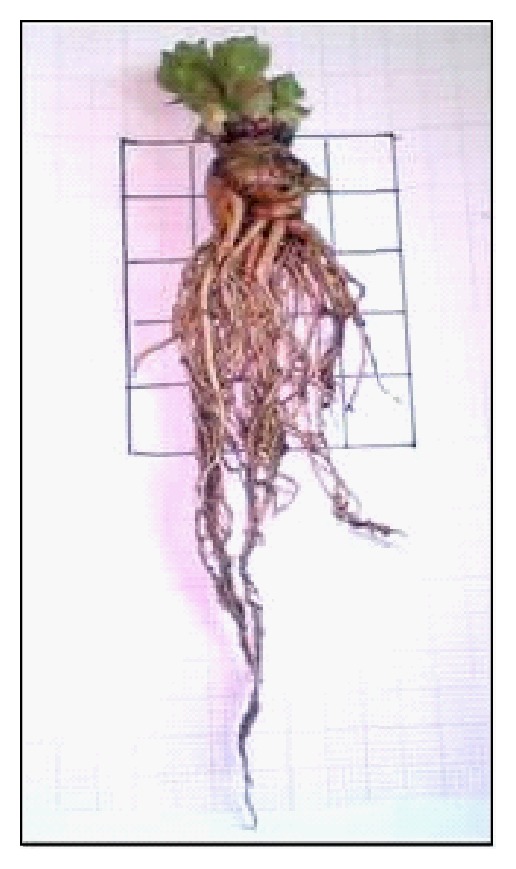
Roots and rhizomes of *in vitro* regenerated plant cultivated two years in the green house [79].

*In vitro* obtained plants were phytochemical analyzed for the presence of biologically active substances which were not found in the roots of one-month-old regenerants in test tubes (unpublished data). However, salidroside was produced in the rhizome and roots of one- and two-year old regenerants grown in the mountains [78] and in the green house (Figure 9) (unpublished data). The results were promising as far as salidroside content of regenerants was higher than that of the wild plants.

**Figure 10:**
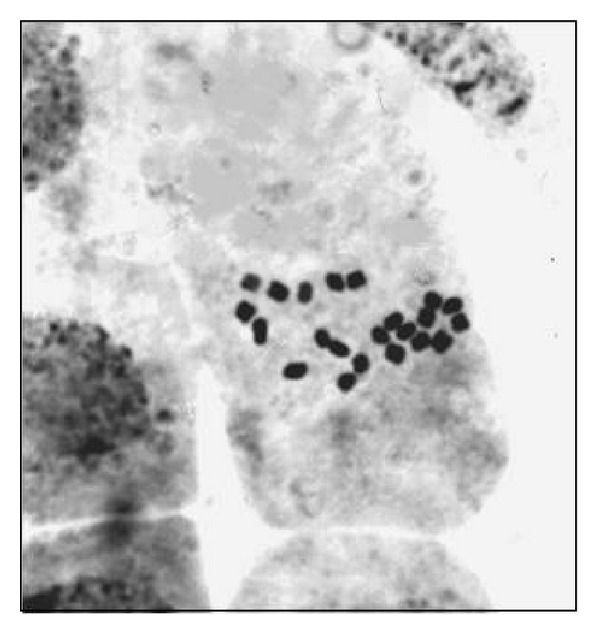
“Squash” preparation of meristematic cells of root tips excised from *in vitro* plant regenerants of *Rhodiola rosea* having diploid chromosome number of 2*n* = 22 [79].

*In vitro* plants obtained on different culture media were subjected to cytological analysis. However, procedures for squash preparations were modified to be applicable to meristematic cells of root tips of *Rhodiola rosea* regenerants. The chromosome number in all samples was 2*n* = 22, which confirmed the diploid level of plant regenerants and cytogenetic identity with the wild-type (Figure 10) [79]. Concerning flowers, leaves, stem, and rhizomes no differences were recorded between the wild plants and the regenerated plants grown for 1 and 2 years *ex vitro* in the adaptation room, in the green house, and in the mountain.

As a result of the experiments efficient schemes for regeneration and micropropagation of *Rh. rosea *were established, despite the difficulties working with wild species. The different schemes for efficient micropropagation, utilizing a range of media and several types of explants, allow flexibility in the investigations. These schemes varied in time duration, labor consumption, and costs.

A recommendable protocol could be the following one:

1. seed decontamination by EtOH for 3 min followed by 20% v/v bleach for 15 min and germination on MS media containing 5–100 mg/L gibberellic acid;
2. shoot multiplication from leaf nodes from seedlings or regenerants on MS enriched either with 2 mg/L zeatin, 0.2 mg/L IAA, and 1000 mg/L casein hydrolysate or with 0.2 mg/L zeatin and 0.2 mg/L IAA (or for a longer period on MS with 1 mg/L BA and 0.1 mg/L IAA or 0.5 mg/L BA and 0.1 mg/L); rooting of plantlets on MS with addition of either 0.2 mg/L IAA, 2.0 mg/L IBA, 0.4 mg/L GA_3_ or 0.2 mg/L IAA, 2.0 mg/L IBA, 0.1 mg/L GA_3_;
3. *in vivo* adaptation in a combination of perlite, peat, and soil in ratio of 1 : 1 : 3.

The whole process could take about three months. For this period more than 100 regenerated plantlets could be propagated from one seedling of rose root. Each regenerant could give three to five explants for further clonal propagation.

Remarkable success of this work was the adaptation of *in vitro* obtained regenerants to the wild high mountain conditions which was not reported by others. This success allows restoration of the habitats and conservation of germplasm of the endangered medicinal species rose root. Accumulation of biomass by micropropagation and salidroside synthesis by *in vitro* regenerants confirm the potential of this ecology friendly biotechnological method for the production of valuable substances from* Rhodiola rosea*. The present conclusions were proved by authors' further phytochemical analysis of salidroside, rosavin, rosin, and rosarin by HPLC detection in roots and rhizomes of *in vitro* plants, reintroduced in nature (unpublished data).

## 4. Potential of Biotechnological Methods for Increasing Biologically Active Substances Production by *Rhodiola rosea*

The plants are traditional source of quite a big number of biologically active substances used in the pharmaceutical industry. The most valuable phytochemicals are products of the secondary metabolism. Most of them have complicated structure which makes their chemical synthesis very difficult and even impossible. Plant *in vitro* cultures are able to produce and accumulate a lot of substances esteemed by the pharmacy and the medicine.

After establishment of *in vitro* cultures, research continues for their implementation in practice. Plant *in vitro* cultures offer possibilities for production of valuable secondary metabolites in bioreactors, for manipulation of the metabolic pathways and metabolic engineering, and for biotransformation. The latter is one of the key mechanisms for improvement of biologically active substances production in *Rhodiola rosea* callus and cell suspension cultures. However, there are not so many reports on *Rhodiola in vitro *cultures used for a production of biologically active substances and for a determination of the optimal parameters for their synthesis *in vitro*.

The possibilities of cell suspension cultures initiated from leaf explant callus were studied [80] for increasing the synthesis of rosavin and other cinnamyl glycosides after addition of transcinnamyl alcohol (optimal concentration of 2.5 mM) into the liquid MS medium. For about 3 days more than 90% of the alcohol was transformed by the cells into various unidentified products. However, one of them, 3-phenyl-2-propenyl-O-(6′-O-*α*′-L-arabinoryranosyl)-*β*-D-glucopyranoside, found in the intracellular spaces, was defined as potential rozavin by very precise methods. Both green and yellow strains of cell cultures were able to glycosylate trans-cinnamyl alcohol into rosavin with yield of 0.03 to 1.01% cell dry weight.

The effect of different precursors of biologically active substances on the biomass and the metabolite production was studied in *Rhodiola* compact callus aggregates in liquid medium [81, 82]. Cinnamyl alcohol concentrations up to 0.1 mM in media did not bring to a significant deviation from the control; 2 to 5 mM changed slightly callus color from dark to light green. In these cultures rosin content was elevated to 1.25% dry weight while rosavin was 0.083% dry weight. Cinnamyl alcohol induced synthesis of four new products, too. Tyrosol from 0.05 mM and 2 mM did not influence callus growth while concentrations of 3 mM up to 9 mM caused decrease in biomass production. Two mM of tyrosol were the optimal levels for salidroside production reaching 2.72% dry weight. Addition of glucose had no positive effect on salidroside accumulation but doubled the rosin production.

Callus tissues cultivated on solid media could produce active substances characteristic for the species [83]. Addition of yeast extract in the media doubled salidroside content (from 0.8% to 1.4) and was twice as high as in five-year-old roots of the intact plants. In the later experiments [84] callus induced from axillary buds or from seedling hypocotyls transformed exogenous cinnamyl alcohol into rosin. However, the biotransformation process was more efficient in the hypocotyl callus where the application of 2.5 mM cinnamyl alcohol resulted in the increase of rosin content up to 1056.183 mg/100 g on solid medium and 776.330 mg/100 g in liquid medium. Callus tissue obtained from axillary buds and treated in the same way produced rosavin in a higher concentration of 92.801 mg/100 g and reached 20% of the amount produced by roots [85]. The extract enriched in rosavins (after transformation) showed the inhibition of behavioral activity of the tested animals—rats.

**Figure 11:**
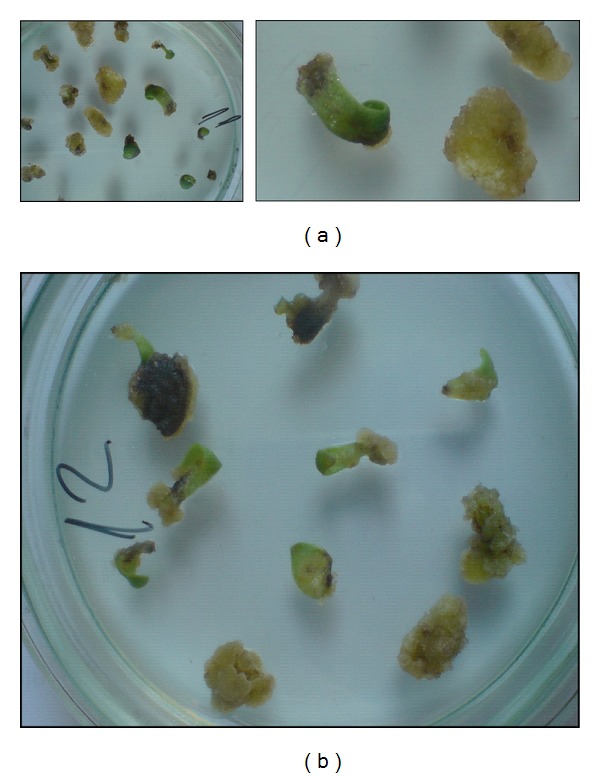
Callus cultures cultivated for 40 days on different media: variant (a) MS medium enriched with 1 mg/L BAP, 0.5 mg/L NAA and 1000 mg/L casein hydrolysate; variant (b) MS medium containing 0.5 mg/L BAP and 1 mg/L IAA.

Induction of callogenesis was successful [86] from leaf explants, isolated from *in vitro* propagated plants, on MS media enriched with BAP in concentration from 0.5 mg/L to 2.0 mg/L; 2-iP—0.3 and 3.0 mg/L; 2,4-D—from 0.1 to 2.0 mg/L; IAA—0.2, 0.3 and 1.0 mg/L; NAA—0.5, 1.0, 1.5 mg/L; glutamine—150 mg/L and casein hydrolysate 1000 mg/L. The highest response to formation of callus (62.85% and 73.17%) was observed on two media-containing 1 mg/L BAP and either 1 mg/L or 0.5 mg/L 2,4-D (Figure 11).

**Figure 12:**
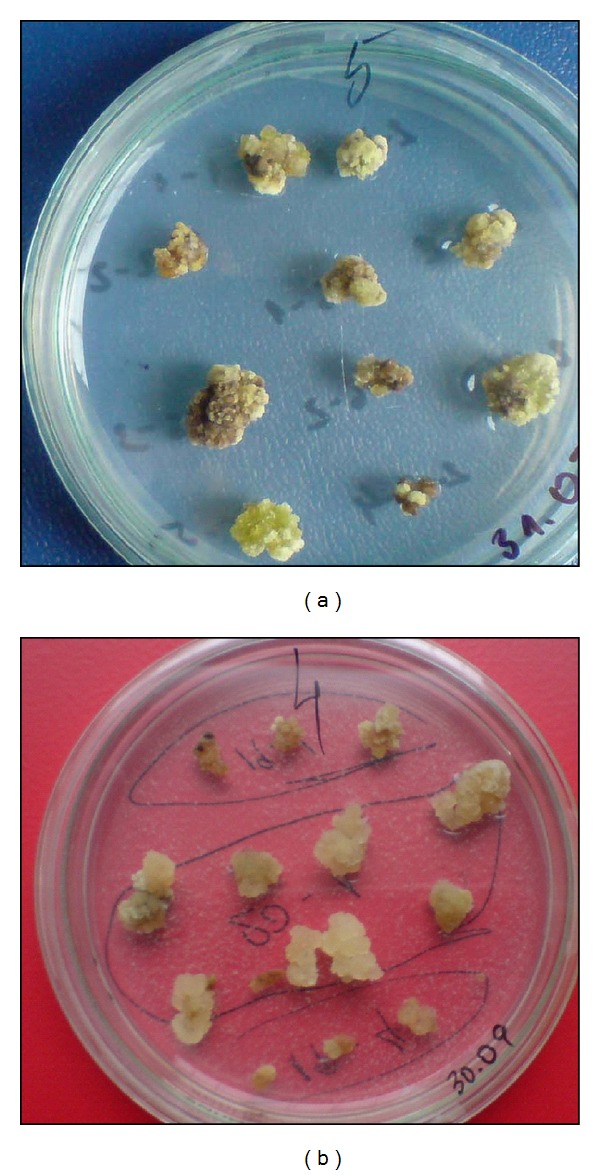
Callus growth after the 3rd passage on different media: variant (a) MS medium enriched with 0.5 mg/L BAP and 1 mg/L 2,4-D; variant (b) MS medium enriched with 1 mg/L BAP and 0.5 mg/L 2,4-D.

Callogenesis was not observed in any of the variants containing 2-ip and most of the variants containing NAA. In this pioneer study optimal combinations and concentrations of phytoregulators were determined for efficient induction and maintaining of callus amenable for long-term cultivation (6 months) (Figure 12).

Total phenolic/flavonoid content and radical scavenging activity was determined in calli. Antioxidant properties were influenced by the composition of the cultivation media. Relationship between the callus color/structure/texture and the secondary metabolite amounts was not significant. Linear correlation between the total phenolic/flavonoid content and the scavenging activity was observed. Bioinformatics prognosis for the optimal recipes of the nutrition media was made based on the analysis of the data obtained in the biological experiments (unpublished data). This study is a pioneering one concerning establishment and maintaining of Bulgarian golden root callus cultures and determination of their phytochemical properties and could be a basis for *in vitro* metabolic engineering and biotransformation for alternative production of valuable substances.

## 5. *In Vitro* Cultures of Rhodiola Genus

Genus Rhodiola consists of 200 polymorphic species. However, less than 20 Rhodiola species including *Rh. alterna*, *Rh. brevipetiolata*, *Rh. crenulata*, *Rh. kirilowii*, *Rh. quadrifida*, *Rh. sachalinensis*, and* Rh. sacra *have pharmacological importance for production of medications [20]. All species of the genus contain different quality of salidroside—one of the basic components of the biologically active complex. Biotechnological research focuses mainly on the species distinguished for their high levels and quality of the substances, valuable for the pharmacological industry. Targets of the experiments are induction of callus and organogenic cultures followed by plant regeneration aiming at alternative *in vitro* synthesis of the desired metabolites and at development of efficient systems for micropropagation for conservation of the species.

The possibility for induction and maintaining of callus cultures was studied [87] in *Rhodiola quadrifida*, a species used in traditional Chinese medicine for more than 1000 years. Callus induction was induced on MS medium enriched with 2,4-D (1.0 mg/L), NAA (2.0 mg/L), BA (0.5 mg/L), and kinetin (0.1 mg/L) and maintained on the same basal medium supplemented with 2,4-D (1.0 mg/L), 6-BA (0.1 mg/L), and kinetin (0.5 mg/L). Analysis of one-month-old callus showed that the nondifferentiated tissues were able to produce salidroside. The authors underlined the role of 2,4-D and BA for production of biologically active substances. Similar observations about the role of the explant, the temperature of cultivation and the pretreatment duration were made in another work [88] on salidroside synthesis in callus obtained from leaf and stem segments of *Rhodiola kirilowii*.

Experiments on callus induction followed by plant regeneration of *Rhodiola coccinea* [89] revealed the importance of callus characteristics for their morphogenic abilities. Only one out of the three callus types were embryogenic. The latter was induced on MS medium containing BA (0.5 mg/L) and IAA (2.0 mg/L). Differentiation and shoot formation was possible by including BA (0.05 mg/L) and IAA (0.5 mg/L) in the medium. Five callus types using different explants from root, stem, leaves, and cotyledons of *Rhodiola sachalinesis *A. Bor. were obtained, too [90]. MS medium containing the phytoregulators BA 3 mg/L, NAA 0.3 mg/L, and temperature of 21–25°C were suitable for callus induction. The same species was an object of other authors [91] who succeeded to induce callus formation (with yellow, green, and red colors) in 88.33% of the leaf explants. BA (2.0 mg/L) and NAA (0.5 mg/L) were also the effective phytoregulators included in MS medium. The authors found out that only green callus was able to form buds on MS medium with lower concentrations of the same phytoregulators BA (1.0 mg/L) and NAA (0.1 mg/L). However, induction of organogenesis was at comparatively low level (21.33%) and needed longer period of time of 50 days. Despite this regenerated plantlets were rooted on half strength MS medium. Cryopreservation of calli induced from seedling explants of the same species* Rhodiola sachalinesis *was successful achieved [92]. The recovery of fresh and green tissues was possible for 6 weeks on nutrient medium containing BA 2.0 mg/L and NAA 0.5 mg/L. *Rhodiola sachalinesis* was an object in the sophisticated experiments of obtained protoplast cultures [93]. Mesophyll protoplasts were isolated from leaves of *in vitro* regenerated plants. The protoplasts which regenerated cell wall, divided to form colonies and calli for a period of 40 days on MS medium enriched with 2,4-D—1.0 mg/L, zeatin—0.5 mg/L, 0.5 M/L mannitol, and casein hydrolysate 500 mg/L were transferred to poorer medium containing only BA and NAA (1.0 mg/L and 0.1 mg/L, resp.). Organogenesis was induced and buds appeared after the p-calli. The shoots developed formed roots on half strength MS medium.

System for *in vitro* cultivation of *Rhodiola sachalinensis* callus aggregates for production of salidroside was established [94]. Its biosynthesis was in standard levels, making the system promising for alternative production of the valuable metabolite.

The possibility for *in vitro* propagation of four *Rhodiola* species—*Rh. crenulata*, *Rh. yunnanensis*, *Rh. fastigata*, and *Rh. sachalinesis* using two types of explants was studies [68]. Stem explants were more suitable for bud induction from *Rh. crenulata*, while leaf material was more efficient for organogenesis in the other three species. The genotype and the phytohormones were important for the efficiency of the regeneration. The optimal combination of phytoregulators for organogenesis in* Rh. crenulata *and* Rh. yunnanensis* was 2.5 mg/L BA and 0.1 mg/L NAA which stimulated bud formation in 71% and 84% in *Rh. crenulata *and* Rh. yunnanensis*, respectively. The medium containing the higher concentrations of NAA (0.5 mg/L) and BA (2.5 mg/L) was more appropriate for *Rh. fastigata* and* Rh. sachalinesis* bud formation 80% of the cases. The shoots from the last two species formed roots easily (87% and 73%, resp.) on medium containing IBA. Regenerants from *Rh. fastigata* were successfully adapted for further *in vivo* development in soil reaching survival rate of 66%.

*In vitro* cultures of *Rhodiola crenulata *aiming at establishment of technology for *in vitro* multiplication of the species were developed [95]. Different explants from wild-growing plants were cultivated on MS basal media with various combinations of plant growth regulators: leaf discs on BA 2.0 mg/L and IAA 0.2 mg/L; flower buds on 2.0 mg/L kinetin; stem nodes on 2.0 mg/L BA and 1.0 mg/L IAA; stem explants on 2.0 mg/L kinetin and 1.0 mg/L IAA. Leaf explants proved to be the most suitable for *in vitro* development with 100% shoot induction on MS medium containing 0.2 mg/L NAA. The other explant types did not show any development for the adequate period of time. The authors put the basis for micropropagation of *Rh. crenulata*.

Accumulation of biomass through micropropagation and the production of biologically active substances in *in vitro *cultures confirm the potential of this environmentally friendly technology to be used for production of *Rhodiola *valuable substances.

## 6. Conclusion

Plant biotechnologies of *Rhodiola rosea *and other* Rhodiola *species have marked considerable success for their short historical period of 20 years. The first investigations on the factors important for the initiation of *in vitro* cultures were deepened and broadened and led to the establishment of complete schemes for micropropagation and systems for production of biologically active substances. During the last years more groups have been involved in the research. The number of examined ecotypes and genotypes of golden root has been increasing and has been covering more *Rhodiola *species. More up-to-date approaches and sophisticated instrumentation have been used which would bring further success to the alternative biosynthesis of valuable *Rhodiola *metabolites and to protection of this precious rare endangered medicinal plant.
